# Gut-brain health effects of PREbiotics in older adults with suspected COgnitive DEcline: design of the PRECODE randomised placebo-controlled trial

**DOI:** 10.3389/fnut.2026.1738622

**Published:** 2026-04-07

**Authors:** Kirsten Kruger, Hieke van der Veen, Maani Beigy, Shauna D. O’Donovan, Natal van Riel, Lianne B. Remie, Esther Aarts, Wilma Steegenga, Paul A. M. Smeets, Mara P. H. van Trijp, Lisette C. P. G. M. de Groot, Yannick Vermeiren

**Affiliations:** 1 Division of Human Nutrition and Health, Wageningen University and Research, Wageningen, Netherlands; 2 Department of Industrial Engineering and Innovation Sciences, Eindhoven University of Technology, Eindhoven, Netherlands; 3 Department of Biomedical Engineering, Eindhoven University of Technology, Eindhoven, Netherlands; 4 Donders Institute for Brain, Cognition and Behaviour Radboud University, Nijmegen, Netherlands

**Keywords:** fMRI, microbiota-gut-brain axis, prebiotics, subjective cognitive decline, working memory

## Abstract

Global lifespan is rising, alongside increasing age-related conditions. Cognitive disorders such as dementia, are projected to triple in number by 2050. Currently, no cure is available for Alzheimer’s disease (AD), the most common form of dementia, and the need for preventative strategies is paramount. Subjective cognitive decline plus (SCD+) lies on the AD continuum, with increased risk of accelerated cognitive decline, providing an ideal target group for early intervention. The microbiota-gut-brain axis (MGBA) is an emerging avenue for prevention, as dietary fibres may beneficially modulate microbiota and microbial metabolites, such as short chain fatty acids and tryptophan-indoles, resulting in improved gastrointestinal and cognitive functioning. PRECODE is a four-armed, randomised, double-blinded, placebo-controlled trial in 164 older adults (60-79 years) with SCD+ and additional “LIfestyle for BRAin Health” (LIBRA) risk factors. The study aims to investigate 26 weeks of supplementation with chicory inulin, resistant dextrin, and seaweed polysaccharide compared to placebo (maltodextrin) on the MGBA. The primary outcome is effects on neurocognition, assessed by brain activation during working memory load measured with blood-oxygen level dependant functional magnetic resonance imaging (BOLD fMRI) and performance during 2-back task. The secondary outcomes include cognition by neuropsychological test battery, brain and intestinal health parameters, and immune and metabolic markers. Findings may guide new preventative modalities in preclinical AD and provide mechanistic insights into the MGBA in individuals at risk for cognitive decline.

**Clinical trial registration:**

https://clinicaltrials.gov/study/NCT06433037

## 1 Introduction

Life expectancy is on the increase globally, however, this increase is not paralleled by greater health span, but rather by age-related conditions such as dementia (1, 2). Estimates indicate that by 2050, the prevalence of dementia will have doubled in Europe and tripled worldwide, with numbers expecting to rise from 50 to 150 million (3–5). Dementia, with Alzheimer’s disease (AD) as its commonest form, is an umbrella term for several neurological conditions that reflect the gradual loss of memory, executive functioning, behavioural conduct and quality of life at old age, ultimately affecting activities of daily living (6). On the AD continuum lies subjective cognitive decline (SCD) and mild cognitive impairment (MCI) (7, 8). Individuals who report experience of decline in cognitive function while performing within the normal range on cognitive tests, are categorised as having SCD (8), while MCI requires the presence of SCD in combination with impaired cognitive testing, alongside preserved functional ability (7).

AD is a devastating condition, with an average disease duration of 4–8 years (9). Despite numerous research efforts, no significant advances have been made into disease-modifying treatments that may attenuate cognitive decline and the accompanying loss of quality of life. Furthermore, treatments targeting key pathological features such as amyloid-beta (Aß) plaque removal by mononuclear antibodies have either failed to reach their primary clinical endpoints, or come with a high-side effect and cost burden (10–13). These drawbacks have made it highly apparent that alternative modalities should be investigated, and specifically, that the focus should shift towards prevention rather than curative pharmacological treatment (1).

The PRECODE study design presented here, aims to address this shift, with a focus on potential preventative nutritional strategies in older adults at risk for cognitive decline. One of the most promising leads in prevention of cognitive decline relates to the microbiota-gut-brain axis (MGBA) (14), which remains underexplored in the context of age-related neurodegeneration.

### 1.1 The microbiota-gut brain axis

**Figure 1:**
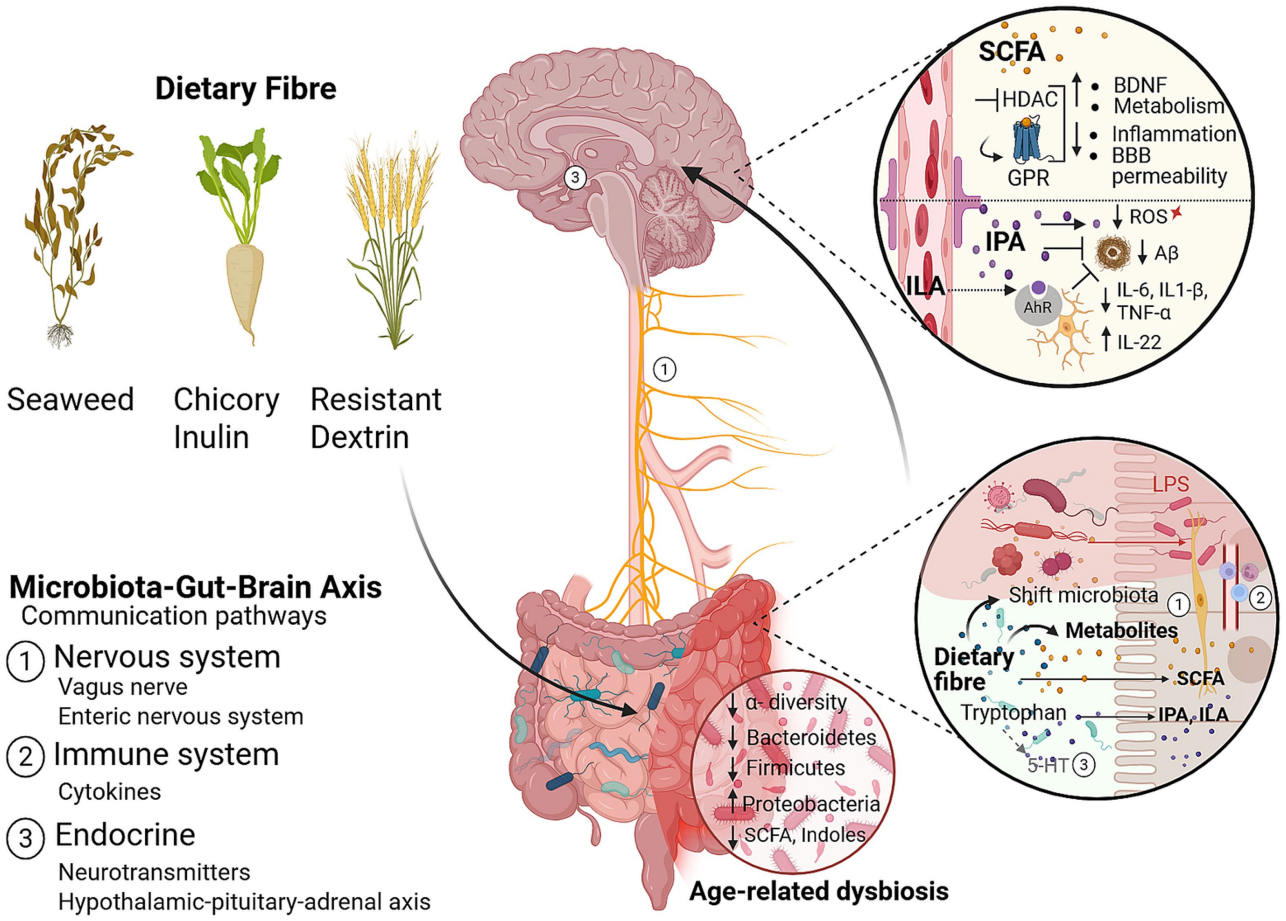
Expected mechanisms by which dietary fibres may prevent age-related cognitive decline along the microbiota-gut-brain axis (MGBA). The MGBA is bidirectional communication system involving neuronal, immune, and endocrine pathways. Commensal microbiota and -derived metabolites modulate components along the axis that influence cognition, such as neurotransmitter production, vagus nerve function, and stress responses (14, 15, 243). Age-related microbial dysbiosis leads to compositional and functional microbial deficits. Chicory inulin, resistant dextrin and (seaweed) ß-glucans may restore deficits by increasing beneficial microbiota such as *Bifidobacteria*, *Parabacteroides* and *Lactobacillus* (16, 142, 149). Dietary fibre consumption modulates the production of microbial metabolites, e.g., short-chain fatty acids (SCFAs), and indoles, which improve cognitive function by modulating BBB permeability, neuroinflammation, and neurogenesis. SCFA exert their effects on cognition through G-protein coupled receptor activation (GPR41, GPR43), histone deacetylase (HDAC) inhibition, and increased brain derived neurotrophic factor (BDNF) expression (80, 81). Beneficial indoles such as indole propionic acid (IPA) and indole lactic acid (ILA) reduce neuroinflammation by aryl hydrocarbon receptor (AhR) activation, exert potent anti-oxidant effects by neutralising hydroxyl radicals, and prevent amyloid-β plaque formation- a hallmark of Alzheimer’s disease pathology (82, 211, 247–249, 253). ROS, Reactive oxygen species; IL, Interleukin; TNF-a, Tumour necrosis factor alpha; LPS, Lipopolysaccharide; 5-HT, 5-Hydroxytryptamine. Figure created with BioRender.com and exported under a publication license (CC BY 4.0).

The MGBA is a bidirectional communication system that includes neural, endocrine, immunological and metabolic signalling between the gut and brain (15) (Figure 1). Modification of microbiota and its metabolites has been associated with cognitive improvements in various preclinical (16–18) and clinical trials (19–25). Specifically, these trials were able to modulate the microbiota and metabolites through nutritional interventions, such as dietary fibre supplementation.

The gut microbiota is involved in various key processes such as nutrient absorption and biosynthesis, immune modulation, and enteric and central neurotransmission (26, 27). With ageing, microbiota composition and function shifts towards a more dysbiotic state (28–30), with reduced microbial diversity, lowered abundance of beneficial bacteria, and a rise in pathobionts (30, 31). Dysbiosis of the microbiota, alongside age-related decline in barrier function are associated with increased inflammation and permeability of the intestinal epithelial barrier (32–35). This impairment may result in excessive translocation of bioactive molecules and endotoxins such as lipopolysaccharide (LPS) (36, 37), triggering systemic inflammatory responses and neuropathological features (38, 39). Similarly, the blood brain barrier (BBB) becomes more permeable (40–42). Altered BBB permeability may play a role in neuroinflammation (40), which in turn contributes to neural degeneration (43) and increases the risk for neurocognitive impairment seen in SCD, MCI and AD (7, 44–46).

Preclinical studies have strengthened the role of the MGBA in cognitive decline. For example, faecal microbiota transplants from AD patients to rodents induced AD-like behavioural impairments, alongside reduced neurogenesis- demonstrating a causal role of the gut microbiome in AD (47). Furthermore, animal studies with induced microbial infections, antibiotic and probiotic treatment, all highlighted mechanistic associations with gut microbiota in cognition and AD-related pathogenesis (27, 48–50). Clinical studies relating to the MGBA along the AD continuum are, however, still limited, with a single study performed in SCD (51), and a handful in MCI (52, 53) and AD (27, 54, 55). These studies highlight differences in key commensal bacteria compared to aged-matched controls. In SCD specifically, changes in anti-inflammatory profiles were observed, such as reduced *Faecalibacterium* abundance (51).

Microbiota composition is most strongly modified by diet across our lifespans (56). Diet is also a modifiable risk factor for AD, and therefore gut-targeted dietary interventions may mitigate AD risk (57). In this context, dietary fibres and prebiotics are emerging as key modulators of the MGBA and show promise in age-related cognitive deterioration (58, 59).

### 1.2 Dietary fibre and prebiotics along the MGBA for cognitive functioning

Prebiotics are defined by the International Scientific Association for Probiotics and Prebiotics (ISAPP) as “substrates that are selectively utilised by host microorganisms, conferring a health benefit” (60, 61). Most prebiotics are dietary fibres- plant-based carbohydrates that are resistant to host digestion in the intestinal tract (61, 62). However, not all dietary fibres are prebiotics; only fibres with proven health benefits through microbiota modulation *in vivo* are considered prebiotics (60). Unlike probiotics, which involve introducing microbial strains, prebiotics work by stimulating the growth and activity of beneficial bacteria already present in the gastrointestinal tract (60, 63).

In terms of health benefits, dietary fibres have shown to improve glucose homeostasis (64), insulin sensitivity (65), lipid profiles (66), and lower systemic inflammatory markers (67, 68). Beneficial effects of fibres on the gut include intestinal inflammation (69, 70), lowered faecal pH (71, 72), regulation of bowel movements, improved intestinal transit time (ITT) and stool consistency (64, 73, 74). Additionally, evidence for a positive impact of dietary fibres on cognitive functioning is increasingly recognised (75, 76).

One of the main mechanisms by which prebiotics may confer health benefits along the MGBA is by influencing the production of metabolites- considered the functional effectors of microbiota (77). Short chain fatty acids (SCFA) are directly produced from microbial carbohydrate fermentation (78), while dietary fibre indirectly regulates microbial tryptophan metabolism, shifting towards beneficial indole production (79). SCFA and beneficial indoles, such as indole propionic acid (IPA) can cross the BBB, where they elicit neuroprotective effects by epigenetic regulation, modulating neuroinflammation, regulating BBB integrity, optimising brain metabolism, and inhibition of amyloid protein formation (80–83). In the gut, both SCFA and indoles maintain intestinal barrier function (82, 84). Studies indicate that both SCFA and indole levels decrease with age, likely due to age-related microbial deficits (85, 86).

Inulin, resistant dextrin and *β*-glucans are dietary fibres that have all demonstrated beneficial effects on the MGBA in preclinical and clinical studies, and for this reason have been included as our investigational products (17, 20, 87). These effects include, amongst others, beneficial modulation of microbiota composition (88, 89), amelioration of microbial dysbiosis (17), increased SCFA production (72, 90, 91), and improved intestinal barrier function (20, 92). In the brain, reported effects include amelioration of neuroinflammation (93, 94), improved mood (21) and cognition (23, 95, 96), and enhanced overall mental health scores (97). Additionally, dietary fibre intake declines with ageing, due to factors such as mastication difficulty, anorexia of ageing, sensory and food preference changes and increased gastrointestinal sensitivity (98–100). Supplementation in older adults may therefore have the potential to offset age-related deterioration in gut and brain function.

### 1.3 Cognitive ageing, SCD and lifestyle risk factors

Physiological cognitive ageing is associated with gradual neuronal losses in the hippocampus, temporal cortex, and prefrontal cortex (101–103). Such structural and functional connectivity changes are associated with decreased cognitive performances in executive functioning and working memory, identifying them as key regions of interest (104–109). Additionally, executive function and working memory are some of the earliest domains affected by AD (110), and predict progression from MCI to AD (111). Compensatory mechanisms occur in SCD and preclinical AD, and although individuals with SCD typically perform within normal ranges on cognitive tests, prior studies have reported subtle impairments in memory and executive functioning compared to cognitively healthy controls (112–114). In particular, fluctuating deficits in short-term working memory were noted (115). Working memory is a specialised, short-term active storage system that underlies a range of higher-order functions including reasoning, decision-making, judgment, and language comprehension (116). Given its central role in executive functioning and its susceptibility to early cognitive changes, working memory is a key domain of interest.

**Figure 2:**
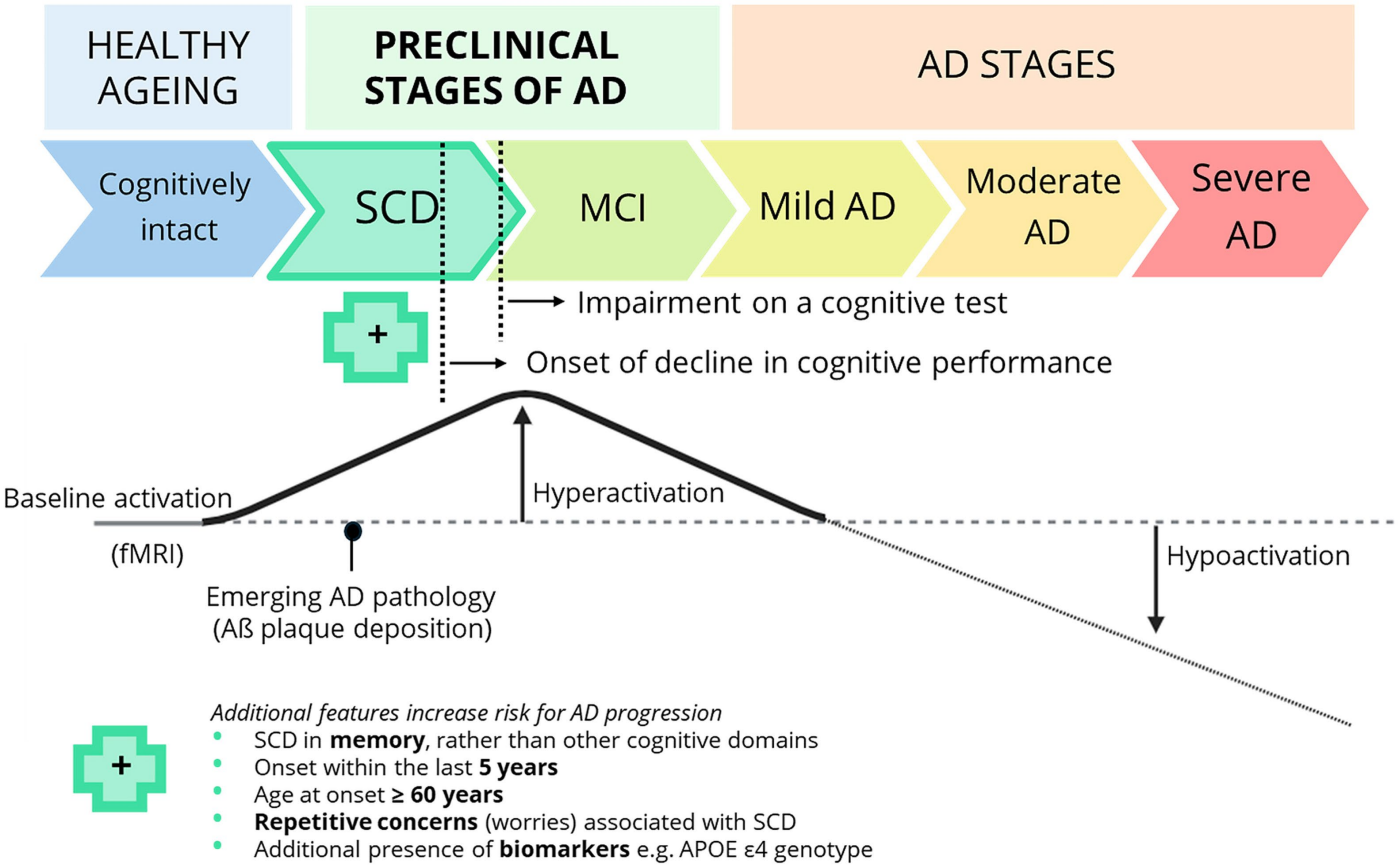
Subjective cognitive decline plus (SCD+) and cognitive changes along the continuum of Alzheimer’s disease (AD). SCD lies within preclinical stages of AD and precedes an impairment in cognitive testing as observed in mild cognitive impairment (MCI). Individuals with SCD + have additional features that increase the likelihood of progression to AD, such as onset within the past five years, onset after age 60, memory-specific and persistent concerns. The presence of the apolipoprotein E (*APOE*) ε4 genotype, and AD biomarkers (e.g., amyloid-β) further increase the risk for disease progression (113). AD biomarkers correlate with working memory task-related hyperactivation measured with fMRI, reflecting early neurodegenerative and compensatory changes in preclinical AD (118). Load-dependent paradigms such as the *n*-back task allow for the detection of subtle cognitive impairments in SCD (117, 118). SCD may occur up to 10 years before a dementia diagnosis (114), making it an ideal target population for preventative strategies. To date, no studies have examined the effects of dietary fibre in SCD+. Image adapted from Corriveau-Lecavalier et al. (118).

The n-back task is a validated tool to assess working memory capacity and elicits working memory load-dependant activation which can be measured with the use of blood-oxygen-level dependent (BOLD) functional magnetic resonance imaging (fMRI) (117). Changes in this brain activation are detectable in early AD and reflect compensatory mechanisms and possible underlying neuropathology such as Aß plaque deposition (114, 118). Specifically, hyperactivation of working-memory load dependent brain regions is seen, compared to healthy controls (118). As AD progresses and compensatory mechanisms fail, these regions start demonstrating hypoactivation (Figure 2). The n-back task performed during fMRI has also shown sensitivity to distinguish individuals with SCD from healthy controls (117, 119).

These subtle cognitive deviations, particularly in working memory, highlight SCD as a target group for identifying individuals at increased risk for AD. SCD refers to self-perceived worsening of cognitive functioning in the absence of objective impairment on standardised assessments, relative to age- and sex-matched norms (113). SCD plus (SCD+) has been further defined to include additional features associated with an increased risk of progression to AD (Figure 2). To further identify individuals at increased risk, our study utilises risk factors from the “LIfestyle for BRAin Health” (LIBRA) score as part of the inclusion criteria. The LIBRA score captures a range of modifiable lifestyle-related risk factors, including hypertension, obesity, diabetes, hypercholesterolemia, and coronary heart disease (41), which have been shown to predict variance in cognitive function and dementia risk (57).

The PRECODE study aims to investigate the effects of three (potential) prebiotic dietary fibres, chicory inulin, resistant dextrin, and seaweed-derived *β*-glucans, on the MGBA in older adults with SCD+. The combination of SCD + features alongside lifestyle risk factors, allows for a study population that is both at risk and likely to benefit from early interventions targeting the MGBA.

## 2 Methods and analysis

### 2.1 Study aim and objectives

The aim of the PRECODE study is to evaluate the effect of 26 weeks of inulin, resistant dextrin and seaweed fibres supplementation compared to placebo (maltodextrin) on the MBGA in older adults with SCD+, with the following objectives (Table 1):

- *Primary objective*: To determine the effect of 26 weeks of inulin, resistant dextrin and seaweed fibre supplementation on cognitive function by evaluating brain activation during working memory load measured with BOLD fMRI and task performance during 2-back task.
- *Secondary objectives*: To determine the effects of 26 weeks of inulin, resistant dextrin and seaweed fibre supplementation on (1) cognition as measured by a neuropsychological test battery (NTB) (2) other relevant brain health parameters (3) relevant gut health parameters and (4) immune and metabolic parameters (Table 1)
- *Tertiary objectives*: To determine the effects 26 weeks of inulin, resistant dextrin and seaweed fibre supplementation on (1) physiological parameters and (2) mood.

**Table 1:** Objectives and outcome measurements.

Primary objective	Primary outcome measure	
To evaluate the effect of 26 weeks of inulin, resistant dextrin and seaweed fibres supplementation compared to placebo (maltodextrin) on the microbiota-gut-brain axis in older adults with SCD+ on:		
1. Cognitive functioning	i. Brain activation during working memory load measured with BOLD fMRI and ii. task performance during 2-back task	

Secondary objectives	Secondary outcome measures	
To evaluate the effect of 26 weeks of inulin, resistant dextrin and seaweed fibre supplementation compared to placebo (maltodextrin) in older adults with SCD+ on:		
1. Neuropsychological test battery (NTB)	Cognitive functioning as measured by a NTB	CFC scores
2. Other relevant brain health parameters	In plasma/serum	
	Tryptophan metabolites	
	Aβ1-42/Aβ1-40 ratio	
	NfL and GFAP levels	
	Cortisol levels	
	BDNF levels	
	Structural MRI	
	*T*_1_-weighted and FLAIR anatomical images of ROIs	ROIs: Hippocampi, (pre-) frontal, temporal
3. Relevant intestinal health parameters	Faecal microbiota composition	Quantitative ddPCR; Qualitative 16 s rRNA sequencing
	Intestinal barrier integrity	iFABP, sCD14, LBP, LPS, Zonulin
	Intestinal inflammation	Calprotectin, Lipocalin, sIgA, β-defensin
	Gastrointestinal transit time	
	Individual SCFAs and BCFAs	
	Faecal water content and pH	
	GSRS and BSS questionnaires	
4. Immune and metabolic parameters	Inflammatory cytokines; Fasting glucose and insulin; HbA1c	
	Lipogram	LDL, total cholesterol, triglycerides

Tertiary objectives	Tertiary outcome measures	
To evaluate the effect of 26 weeks of inulin, resistant dextrin and seaweed fibre supplementation compared to placebo (maltodextrin) in older adults with SCD+ on:		
1. Physiological parameters		
	Heart rate	Measured by Samsung Active 2.0 Smartwatches
	Physical activity	
	BMI	Height, weight
	Blood pressure	
2. Mood- as determined by		
(i) Wearable Samsung Active 2.0 Smartwatches	Sad, stressed, neutral, happy, or angry	3 hourly mood tracking via push notifications
(ii) Questionnaires	Self-reported depression and anxiety	GDS-15, GAD-7

Aβ, Amyloid-beta; ApoE, Apolipoprotein E; BCFAs, Branched-Chain Fatty Acids; BDNF, Brain-Derived Neurotrophic Factor; BMI, Body Mass Index; BOLD, Blood Oxygen Level Dependent; BSS, Bristol Stool Scale; CFC, Cognitive Functional Composite; ddPCR, Droplet Digital Polymerase Chain Reaction; FLAIR, Fluid-Attenuated Inversion Recovery; (f)MRI, (Functional) Magnetic Resonance Imaging; GAD-7, Generalised Anxiety Disorder-7; GDS-15, Geriatric Depression Scale (15-item); GFAP, Glial Fibrillary Acidic Protein; GSRS, Gastrointestinal Symptom Rating Scale; HbA1c, Glycated Haemoglobin A1c; iFABP, Intestinal Fatty Acid Binding Protein; LBP, Lipopolysaccharide-Binding Protein; LDL, Low-Density Lipoprotein; LPS, Lipopolysaccharide; NfL, Neurofilament light chain; NTB, Neuropsychological Test Battery; ROIs, Regions of Interest; sCD14, Soluble Cluster of Differentiation 14; SCFA, Short-Chain Fatty Acids; sIgA, Secretory Immunoglobulin A; SCD+, Subjective Cognitive Decline Plus.

Exploratory outcomes include amongst others, to determine the effects on gut hormones. Additionally, further baseline and background data will be collected from all participants, including demographic information (age, sex, education), risk factor profile (LIBRA short-form questionnaire), weight, height, and blood pressure. Detailed medical history, current medication use, dietary intake and supplement use will be recorded via structured questionnaires. Frailty will be assessed using a frailty questionnaire and grip strength measurements. For female participants, obstetric and gynaecological history will be gathered at baseline.

### 2.2 Study design and setting

The PRECODE study is a randomised, double-blinded placebo-controlled, four-armed parallel trial in older adults (60–79 years) with SCD+, with a duration of 26 weeks (Clinical trials identifier NCT06433037). Participants (*n* = 164) will be randomised by block-randomisation (based on sex, age, education and risk factor profile) in four different groups of equal size (*n* = 41). Either one of the following will be administered daily:

1. Placebo (Maltodextrin) – 7 g per day, divided over two dosages of 3.5 g.
2. Chicory inulin – 12 g per day, divided over two dosages of 6 g.
3. Resistant dextrin – 14 g per day, divided over two dosages of 7 g.
4. Seaweed polysaccharide – 1 g per day, divided over two dosages of 0.5 g. (Seaweed will additionally contain 7 g (3.5 g per dose) of placebo as a volumetric and isocaloric filler.)

**Figure 3:**
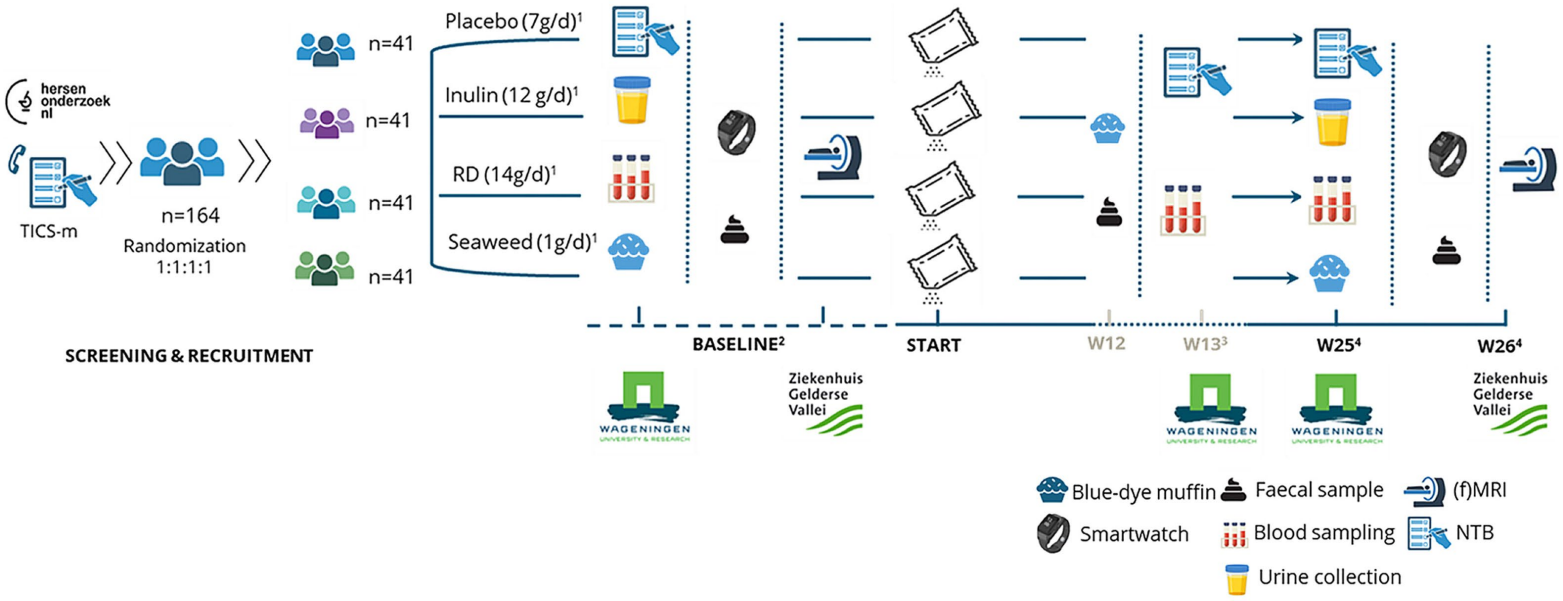
PRECODE study timeline overview. ^1^Will be provided in two dosages per day. ^2^Baseline visits corresponding to WUR *T*_0_ and ZGV *T*_0_ visits. ^3^Week 12 and 13 visits corresponding to *T*_mid_ visits, in a subselection (*n* = 80) of participants only. ^4^Week 25 and week 26 end visits, corresponding to WUR *T*_1_ and ZGV *T*_1_ visits. RD, Resistant dextrin; TICS-m, Modified telephone interview for cognitive status.

Participants will undergo assessments at baseline (*T*_1_), week 13 (*T*_mid_) and at study end (*T*_1_), after 26 weeks at the Division of Human Nutrition & Health (HNH) at Wageningen University (WUR) and the Gelderse Vallei Hospital (ZGV) in the Netherlands (Figure 3). Each participant will have four study visits in total: two at *T*_0_, and two at *T*_1_. In a subselection of participants (*n* = 80), there will be an additional mid-visit (*T*_mid_) in week 13, with five study visits in total. At *T*_0_ and *T*_1_ participants will first visit WUR for investigations and a week later visit ZGV for fMRI scanning. In the week between the visits, participants will collect two faecal samples, wear smartwatches and complete online questionnaires at home. The *T*_mid_ visit consists of a single visit to WUR only. To ensure compliance and to address any questions or concerns that arise during the intervention, five phone calls (10–15 min) will be made to participants during the course of the study.

### 2.3 Study population

To evaluate the potential preventative effects of the investigational products, our study population consists of individuals with SCD + and at least two additional lifestyle-related risk factors for cognitive decline, as outlined in Table 2. To be eligible for participation, subjects must meet all of the inclusion criteria. An individual who meets one or more exclusion criteria will be excluded from participation.

**Table 2:** Inclusion and exclusion criteria.

Inclusion criteria	Exclusion criteria
1. Written informed consent	1. Current participation in other intervention trials
2. Fluency in Dutch (speaking, reading, writing)	2. Technologically illiterate (complete incompetence in working with computers, apps, online questionnaires, smartwatches)
3. Age between 60–79 years (at screening)	3. No internet access from home
4. Subjective cognitive decline plus (SCD+), (criteria of Jessen et al. (113)): 4.1 Self-reported worsening of memory; 4.2 Indication of repetitive concerns (worries) associated with SCD; 4.3 With at least one of the following two features present: (i) onset of SCD within the last 5 years; (ii) age at onset ≥60 years of age	4. Clinical diagnosis of ≥1 of the following: Neurological pathology (e.g., MCI, dementia, multiple sclerosis, Parkinson’s disease, epilepsy) Current malignant disease(s), with or without treatment Current psychiatric disorder(s) (e.g., major depressive disorder, bipolar disorder, schizophrenia, anxiety, psychosis, post-traumatic stress disorder (PTSD)) Symptomatic/decompensated cardiovascular disease (e.g., stroke, angina pectoris, heart failure, recent myocardial infarction) Severe visual impairment or blindness Hearing or communicative impairment Gastrointestinal tract disorder such as irritable bowel syndrome or inflammatory bowel disease (e.g., Crohn’s disease or ulcerative colitis).
5. Presence of self-reported risk factors for cognitive decline based on the weighted LIBRA criteria. At least 2 modifiable risk factors need to be present: (i) Diabetes mellitus type II^1 (has your doctor ever told you that you have diabetes? yes/no)^ (ii) High cholesterol^1 (has your doctor ever told you your cholesterol is too high? yes/no)^ (iii) Hypertension^1 (has your doctor ever told you that you have high blood pressure? yes/no)^ (iv) High BMI^1,2^ (v) Heart disease^1 (has your doctor ever told you that you have a heart or blood vessel condition? yes/no)^ (vi) Unhealthy diet^1 (lower regular adherence to Mediterranean diet components such as fish, vegetables, olive oil, pasta and red wine)^	5. Current or recent (<6 weeks) use of prebiotic, probiotic, or dietary fibre supplements that may modulate the microbiota, or unwilling to stop the use of supplements during the study
	6. Current or recent (<6 weeks) use of algae/phytoplankton supplements such as spirulina or chlorella, or unwilling to stop the use of supplements during the study
	7. Use of psychotropic medication (anti-depressants, anti-psychotics)
	8. Use of antibiotics in the past 3 months or planned use during the study
	9. Being an employee of the Human Nutrition and Health Division of Wageningen University
	10. Significant cognitive impairment assessed using the Modified Telephone Interview for Cognitive Status battery (TICS-m score <23)
	11. Request to have Apo-E genotype result disclosed
	12. Allergies to fish or shellfish
	13. Having a contra-indication to MRI scanning including: i. Ferromagnetic implants: - Active implantable medical devices such as: insulin pump /medicine pump / neurostimulator; pacemaker / defibrillator; - Other passive implants such as: punctured port-a-cath; synthetic heart valve ii. Intra-orbital or intra-ocular metallic fragments iii. Claustrophobia
	14. Any other relevant medical or surgical history that is not mentioned in above criteria, which could influence the study outcome measurements, as determined by the researchers.

^1^Criteria defined by the short version questions from LIBRA Administration. ^2^Defined as ≥25 kg/m^2^ for 60–69 years old, and ≥28 kg/m^2^ for ≥70 years old, based on self-reported height and weight.

### 2.4 Recruitment, consent, screening and inclusion

Participants will be recruited through Hersenonderzoek.nl (a Dutch online registry of individuals interested in participating in brain health research), the WUR HNH participant database, local outreach efforts, and (social) media advertisements. Eligible individuals who express interest via email will be invited to attend an in-person information session at WUR. Following this session, interested participants may sign informed consent and subsequently, eligibility will be assessed based on the inclusion and exclusion criteria. Hereafter, participants will undergo the Modified Telephone Interview for Cognitive Status (TICS-m). The TICS-m is used for screening purposes and has been validated for use in older adults, with lower scores indicative of potential MCI or AD (<23) (120, 121). Participants will be given a minimum of one week to consider their participation. Participants will be asked for additional consent for their data as well as any remaining bodily material to be stored up to 15 years and used for other research purposes.

### 2.5 Sample size calculation

To date, most fMRI studies investigating gut interventions have focused on emotional processing (122, 123), with few examining working memory (75, 124). To our best knowledge, no studies have evaluated the effects of dietary fibres in older adults with (or without) cognitive impairment or SCD using fMRI-based n-back tasks. Therefore, sample size was calculated based on the effects on memory performance rather than working memory-related BOLD response. However, at the time of designing the study, no study had evaluated the effects of dietary fibres on working memory performance in SCD, which is why our sample size calculations are based on two different study populations (healthy older adults and MCI patients), as the cognitive level of an SCD population could be considered “in between” these two populations. The effect sizes from one dietary fibre (fermented seaweed) (125) and probiotic study (126) were included in the sample size calculation. The included dietary fibre study in healthy older adults demonstrated effects on cognitive function, including working memory (125), while the probiotic study in older adults with MCI showed improvements in immediate memory, visuospatial and delayed memory (126). The Cohen’s f effect sizes were 0.17 and 0.42, respectively, yielding an average effect size of 0.30. Based on this, a total sample size of *n* = 138 was calculated using G*Power (version 3.1.9.7, Universität Kiel, Germany) for an ANCOVA (*α* = 0.05, power = 80%, 4 groups, baseline as covariate). Allowing for an estimated 15% dropout due to fMRI data quality issues and intervention compliance, the final required sample size is *n* = 164 (41 participants per group).

### 2.6 Participant timeline and data collection procedures

All data collection will follow standardised operating procedures with validated tests and standardised tools used for all outcome assessments. The CASTOR electronic data capture (EDC) system (127) will be used for electronic case report forms to minimise data entry errors. Laboratory procedures will be recorded via an electronic journal.

Following confirmation of eligibility and written informed consent, participants will be scheduled for first visits. In preparation, a standardised evening meal, written instructions and questionnaire booklet will delivered to their homes. For all WUR study visits, participants will be instructed to consume the standardised meal the night before their visit and to fast from 22:00 (water and chronic medication permitted).

#### 2.6.1 Baseline visits

##### 2.6.1.1 WUR-*T*_0_

At the WUR study centre fasting urine and blood (35 mL via venepuncture) will be collected, processed (plasma, serum, and whole blood) and stored at −80 °C. After blood taking, participants will receive a standardised breakfast, followed by a NTB and physiological measurements (Tables 3, 4). During waiting periods, baseline questionnaires on demographic information (age, sex, education, cognitive risk factor profile), medical history, current medication, dietary habits, supplement use, and frailty status will be completed in CASTOR EDC (Table 4). Female participants will also provide obstetric and gynaecological history. Participants will then consume two blue muffins for intestinal transit time (ITT) calculation, receive instructions and materials for home faecal sample collection, gut health questionnaires, and a smartwatch with instructions on mood push notification reporting. The visit will last ~3 h.

**Table 3:** Overview of biological and physiological outcome assessments.

Outcome assessment marker		Timepoin*t*^1^		
Brain health markers		*T*_0_	*T*_mid_	*T*_1_
(1) fMRI	BOLD signal changes and task accuracy during a 2-back fMRI task	x		x
	*T*_1_/FLAIR anatomical brain scans	x		x
(2) Blood markers	ApoE genotype (total blood)	x		
	Cortisol (serum)	x		x
	Brain Derived Neurotropic Factor (serum)	x	x	x
	Aβ1-42/Aβ1-40 ratio; Neurofilament Light Chain and Glial Fibrillary Acidic Protein levels (plasma)	x		x
	Key kynurenine pathway neurotransmitters and tryptophan metabolites	x	x	x
(3) Urine	Key kynurenine pathway neurotransmitters and tryptophan metabolites	x		x
(4) Smartwatch	Mood via push notifications	x		x
Gut health assessments				
(1) Questionnaires	Bristol Stool Scale	x	x	x
	Gastrointestinal symptom rating scale (GSRS)^2^	x	x	x
(2) Faecal markers	Qualitative microbiota composition (16 s rRNA sequencing)	x	x	x
	Quantitative microbiota (ddPCR)	x	x	x
	Short and branched chain fatty acids	x	x	x
	pH, water content	x	x	x
	Intestinal inflammation (Calprotectin, Lipocalin, β-defensin, sIgA)	x		x
(3) Plasma markers	Intestinal barrier function (Zonulin, iFABP, LBP, sCD14, LPS)	x		x
	Gut hormones	x		x
Metabolic and immune markers				
Plasma/serum	Vitamin D	x		x
	Inflammatory cytokines	x		x
	HbA1c, glucose, insulin	x	x	x
	Lipogram (LDL, HDL, total cholesterol, triglycerides)	x		x
Physiological assessments				
(1) Clinical measurements	Blood pressure, weight, height, BMI	x	x	x
	Grip strength	x	x	x
(2) Smartwatch	Heart rate	x		x
	Physical activity	x		x
Other questionnaires (not outcome measurements)				
(1) Dietary intake	Food Frequency Questionnaire (MIND-diet adjusted)	x	x	x
(2) Participation perception	Study participation perception			x
(3) Compliance diary		x	x	x
(4) General health questionnaires^3^		x		x
(5) Frailty questionnaire (FRAIL-tool)		x		x

^1^*T*_0_: baseline; *T*_mid_: mid-study visit, week 13 (subselection *n* = 80), *T*_1_: end visit, week 26. ^2^ In addition to completion at the three time points, the GSRS will be completed weekly for the first month, to evaluate responses to increased fibre intake. ^3^Population demographics, e.g., age, sex, risk factor profile (LIBRA), medical history, medication, supplement use, for females: information regarding gynaecological and obstetric history. At *T*_1_ a subselection of questions are filled again to determine any changes in health or medication use over the study period. MIND: Mediterranean-DASH diet Intervention for Neurodegenerative Delay.

**Table 4:** Overview of cognitive assessment questionnaires.

Assessment	Cognitive domains	Completion time	Timepoint^1^			
			S	*T*_0_	*T*_mid_	*T*_1_
Telephonic Interview for Cognitive Status (TICS-m)	Screening tool for cognitive disorders (e.g., MCI, dementia)	30 min	x			
Cognitive Failure Questionnaire	Perception of subjective cognitive decline	10 min		x	x	x
NTB						
Montreal cognitive assessment scale	Testing for light cognitive deterioration and global cognition	15 min		x		
Cognitive functional composite						
ADAS-Cog word recognition	Episodic memory	25–30 min in total		x	x	x
ADAS-Cog orientation	Episodic memory			x	x	x
ADAS-Cog word recall	Episodic memory			x	x	x
Digit symbol substitution test	Executive function			x	x	x
Digit span backward task	Working memory			x	x	x
Category fluency test	Executive function			x	x	x
Mood						
Geriatric depression scale (GDS-15)	Depression	7 min		x	x	x
Generalised anxiety disorder (GAD-7)	Anxiety	10 min		x	x	x

^1^S: Screening; *T*_0_: baseline; *T*_mid_: mid-study visit, week 13 (subselection *n* = 80); *T*_1_: end visit, week 26. ADAS: Alzheimer’s Disease Assessment Scale.

##### 2.6.1.2 Home activities

In the week between the WUR and ZGV visits, participants will collect two faecal samples and record blue stool colour appearance for ITT calculation. The smartwatches will be worn from the evening after the WUR visit until the morning of the ZGV visits. Participants will continue the completion of baseline questionnaires in CASTOR EDC, to be finalised before the ZGV visit.

##### 2.6.1.3 ZGV-*T*_0_

One week following the WUR visit, participants will visit the ZGV study centre for (f)MRI scanning. Participants will hand in their faecal samples, BSS & GSRS questionnaires and smartwatches. In line with scanning protocols participants will complete a MRI screening form, ensuring MRI-related contraindications have not changed since inclusion eligibility checks. Safety procedures include the provision of MRI suitable clothing and spectacles (if needed, based on visual acuity), earplugs to reduce scanner noise, and placement of foam pads to minimise head movement and enhance comfort. Communication with the participant will be maintained via an intercom and an alarm bell will be provided to alert of any issues during scanning. Prior to scanning, participants will complete a 5-min computerised practice session of the n-back task to ensure task comprehension. After completing the fMRI scan, participants will receive supplements for the 26 week intervention (first 13 weeks, in *T*_mid_ subselection group) and a compliance diary. The visit will last ~1.5 h.

#### 2.6.2 Intervention start (week 1)

After the ZGV-*T*_0_ visit, the 26 week intervention starts. Subjects will consume the assigned dietary fibre supplement/placebo during breakfast and dinner. At five occasions, participants will receive a phone call to check compliance, address concerns, and to provide motivation if needed.

#### 2.6.3 Mid-study visits (WUR-*T*_mid_)

In week 13, a subselection of participants (*n* = 80) will visit the WUR study centre for the same assessments and procedures as WUR-*T*_0_, except for urine collection. A standardised meal, faecal sample collection materials and blue muffins will be sent to participants’ homes in week 12. The remaining supplements for the next 13 weeks will be provided after the visit. The visit lasts ~3 h.

#### 2.6.4 End visits (WUR-*T*_1_, ZGV-*T*_1_)

The end visits at WUR-*T*_1_ (week 25) and ZGV-*T*_1_ (week 26) as well as the week between the two visits, follow the exact same procedure as the baseline visits. Standardised meals will be delivered to participants’ homes in week 24. At the final ZGV-*T*_1_ visit, participants will hand in their supplement containers and compliance diaries for adherence verification, and provide feedback on smartwatch use and overall study participation.

### 2.7 Interventions

#### 2.7.1 Rationale for the choice of comparators

To capture a broader spectrum of potential health benefits in the SCD + population, this study includes three distinct dietary fibres, each differing in carbohydrate classification and physicochemical properties (Table 5). Given their structural diversity, varied mechanisms of action and gut-brain effects are anticipated (128). The fibres, and their dosages have been carefully selected based on existing evidence, as is described in section below. To date, none of these studies have been performed in SCD(+).

**Table 5:** Overview of investigational products and dosages.

Intervention	Carbohydrate classification	Dosage	Composition (per 100 g)		Potential Effects
Chicory Inulin	Inulin-type fructan (soluble fibre)	12 g/day (6 g bid)	**Fibre**	**90 g**	↑ SCFAs, ↑ BDNF, ↓ Inflammation, ↑ Memory and Mood, ↑ Beneficial microbiota, ↑ TRP- derived indoles, ↑ Intestinal barrier function
			Energy	839 kJ/ 208 kcal	
			Carbohydrates	97 g (of which sugars 7 g; 90 g fibre)	
Resistant Dextrin	Glucose-oligosaccharide non-digestible (soluble fibre)	14 g/day (7 g bid)	**Fibre**	**82 g**	↑ SCFAs, ↓ Cortisol, ↓ Insulin resistance, ↑ Beneficial microbiota, ↑ TRP-metabolite ratios, ↑ Intestinal barrier function
			Energy	896 kJ/ 220 kcal	
			Carbohydrates	14 g (of which sugars <0.5 g)	
Seaweed polysaccharide (β-glucan and Fucoidan)	Hemicellulose + Non-starch polysaccharide	1 g/day and 7 g/day maltodextrin (500 mg and 3.5 g bid)	**Fibre** ^**1**^	**39.6 g**	↑ SCFAs, ↑ BDNF, ↓ (Neuro)inflammation, ↑ Beneficial microbiota, ↑ Intestinal barrier function
			Energy^1^	1,090 kJ/ 262 kcal	
			Carbohydrates^1^	58.22 g (of which sugars 0.1 g)	
			Fat^1^	1.6 g	
			Protein^1^	26.08 g	
Placebo (Maltodextrin)	Short-chain digestible oligosaccharide	7 g/day (3.5 g bid)	**Fibre**	**0 g**	Used in dietary fibres studies, minimal colonic fermentation
			Energy	1,641 kJ /386 kCal	
			Carbohydrates	96 g (of which sugars 6 g)	

^1^Composition/100 g of seaweed polysaccharide, without addition of maltodextrin.

Inulin as a soluble, non-viscous dietary fibre extracted from chicory roots (129), is recognised as a prebiotic by ISAPP (130), as well as having an European Food Safety Authority (EFSA) approved health claim, relating to gastrointestinal function (129, 131, 132). Resistant dextrin (RD) is also a soluble non-viscous dietary fibre derived from starch that selectively modulate microbiota and offers proven health benefits including blood glucose management (88, 133, 134). Seaweed and macroalgae are emerging as sustainable food sources and contain ß-glucans (135). All three fibres may provide promising novel leads in neurodegenerative diseases (93, 136). Maltodextrin is frequently used as a placebo in human studies and has been used as placebo in inulin, RD and *β*-glucan studies (88, 137, 138).

#### 2.7.2 Evidence summary of investigational products

##### 2.7.2.1 Investigational product 1: chicory inulin

In preclinical studies, inulin and inulin-type fructan supplementation resulted in improved cognitive function and mood with antidepressant and anxiolytic effects, enhanced neurogenesis and synaptic plasticity via upregulation of BDNF signalling, and improved BBB permeability (92, 139). Furthermore, studies demonstrated neuroprotective effects and modulation of neuroinflammation in AD models (140). In terms of gut health, SCFA production and beneficial tryptophan (TRP)-derived metabolites, including beneficial indoles (IPA), were increased (139–141), with reductions in inflammatory cytokines seen in both colon and the brain (92). Changes in gut health parameters, including microbiota composition changes, were associated with beneficial cognitive outcomes in a number of studies, highlighting the role of the MGBA (17, 92, 94, 139).

Consistent with preclinical studies, human studies have also shown beneficial effects of chicory inulin type-fructans (ITFs) on both gut health (19, 89) and cognition (19, 22, 23). Alterations in gut microbiota composition were shown after 1–4 weeks (19, 89, 97) notably, with higher abundances of beneficial *Bifidobacterium* (142). Additional findings include increases in faecal SCFA (91, 143, 144), acute increases in plasma 13C-SCFA (145) and improved intestinal barrier function with changes in tight junctions (146). Specifically, increases in SCFAs were seen in older adults. (144). Effects on brain health have been shown in acute (4 h) or longer term (2–36 weeks) ITF supplementation, including improved episodic memory performance (immediate and delayed) (23), cognitive flexibility (21), mood (19, 21, 23), and overall mental wellbeing (97).

*Risks and benefits*

Inulin is a well-tolerated prebiotic (147). Mild gastrointestinal side effects are reported at higher bolus doses (128, 142). Clinically supported benefits include improved stool frequency (132), glycaemic regulation (148), and emerging brain health effects (23, 76). The European Commission has approved the use of a chicory inulin product for the health claim “chicory inulin contributes to maintenance of normal defecation by increasing stool frequency” (131).

##### 2.7.2.2 Investigational product 2: resistant dextrin

Pre-clinical studies of RD have demonstrated effects on gut health by modulating microbiota composition and increasing SCFA production (133, 149–152). Evidence for cognitive effects remain sparse, with one study showing supplementation of RD or RD combined with maltitol, resulting in improved vigilance and cognitive discrimination performance, independent of glycemic response differences (96). In an *in vitro* study, 7 g given twice daily for 18 days increased total bacterial counts and key butyrate-producing taxa (*Clostridium cluster XIVa, Roseburia*), along with elevated levels of acetate, propionate, and butyrate (150). Similarly, 14 days of RD supplementation in rats increased all three major SCFAs (152). In mice fed a high-fat, high-fructose diet, 10 weeks of RD gavage (5 g/kg) enhanced the abundance of *Prevotella* and *Akkermansia*, reduced serum insulin, and improved lipid profiles. (133).

In clinical studies, RD has demonstrated gut health effects by modulating microbiota composition such as increases in *Bacteroides*, *Parabacteroides* and inhibition of *Clostridum perfringens* (72, 88, 153, 154), while increasing SCFAs (72). Studies also demonstrated a reduction in endotoxemia (LPS) levels (20, 155, 156). In terms of brain health, RD has shown improvement in sleep quality (156), depression and anxiety, as well as reduction of cortisol levels (155, 156), modulation of insulin resistance (20, 155) and alteration of tryptophan metabolite ratios (20, 156). Higher cortisol has been previously associated with poorer overall cognitive functioning and MCI, where altered hypothalamic pituitary adrenal (HPA) axis, is proposed to underly neurotoxicity (157). Insulin resistance has been cited as predictive marker of cognitive decline (158) and beneficial tryptophan metabolites may modulate neuroprotective effects along the MGBA (159, 160).

*Risks and benefits*

Resistant dextrin may potentially cause gastrointestinal discomfort as its main side effect. No other known risks have been demonstrated. Potential benefits of resistant dextrin include gut and brain health effects (88, 150, 154–156), regulation of glycaemic responses and improved insulin resistance, increased satiety and reduction of energy needs (133, 134) Studies show no demonstrable side-effects at high dosages (161).

##### 2.7.2.3 Investigational product 3: seaweed polysaccharide

The investigational product contains *β*-glucan and fucoidan (a seaweed polysaccharide).

Supplementation with both β-glucan and fucoidan individually demonstrated MGBA effects in preclinical models. Gut health effects include beneficial microbiota composition changes (*Bacteroidetes, Lactobacillus, Bifidobacterium, Akkermansia*) (16, 162) increased *α*-diversity (163, 164) and increases in faecal SCFAs (16, 163, 164). Furthermore gut barrier integrity was improved by upregulation of tight junctional proteins (162, 163, 165), strengthening of the mucosal barrier (163, 165, 166) and reduced colonic inflammation (165). Both supplements have demonstrated cognitive effects including improved memory and spatial learning, alongside optimised synaptic and signalling pathways (16, 162, 166). Behavioural changes were also seen with reduced anxiety (164, 167–169). Results indicated that memory function improvement was mediated by increases in BDNF levels (164, 166), and improved insulin signalling (164). Supplement-specific results of *β*-glucan include reduced hippocampal microglia activation (162, 166), improved synaptic ultrastructure (162, 166) and reduced tau hyperphosphorylation (16, 162), while fucoidan has demonstrated neuroprotective effects against Aβ aggregation, plaque formation (167) and Aβ-induced neurotoxicity (170).

In clinical studies, evidence of β-glucan and seaweed effects on the MGBA are still being explored. A recent study of a fucoidan-rich extract derived from *Saccharina latissima* (sugar kelp) in healthy adults, demonstrated increased abundance in beneficial *Bifidobacterium, Faecalibacterium*, and *Lachnospiraceae*, with reduced pro-inflammatory taxa. Further *in vitro* simulations showed dose-dependent increases in SCFAs (87). Additional studies support the effects of fucoidan on intestinal health, with co-supplementation in chronic superficial gastritis resulting in reduced gastric mucosal damage, reduced intestinal inflammation, increased SCFAs, improved gastrointestinal symptoms, and increases in *Bifidobacterium* and *Bacteroides* (171). Studies in prediabetes and *H. Pylori* eradication further demonstrate beneficial effects on microbiota composition as well as reduced systemic inflammation (172, 173). Gastrointestinal benefits of *β*-glucan have also been seen in healthy adults with supplementation demonstrating improved microbiota composition accompanied by increases in SCFAs (174). In terms of cognition, two β-glucan studies demonstrated effects on mood as well as improvements in overall well-being (24, 25), while *Laminaria japonica* (Japanese kelp) enriched with GABA, resulted in improvement in neuropsychological test scores (short-term memory, executive function, intelligence, sensory memory capacity) in senior subjects, with significant increases in BDNF (122).

*Risks and benefits*

No side effects to β-glucans have been reported in clinical trials (137). Daily intake of fucoidan at the intervention dosage has been established as safe (175).

#### 2.7.3 Intervention description

The study has three treatment arms (chicory inulin, resistant dextrin and seaweed polysaccharide) and one placebo arm (maltodextrin). All supplements will be provided in powdered form and will be consumed in the same manner, namely mixed in 200 mL water, coffee or tea, at breakfast and dinner, taken as close as possible to the same time per day. All products and placebo dosages are matched iso-calorically and have been weighed and filled in sachets by a commercial company. Compliance will be monitored by completion of a compliance diary.

To reduce the possibility of side effects the daily dosage is halved, to be taken twice a day. Additionally, to allow for intestinal adaptation to increased fibre intake, participants only consume half of the daily dosage during the first week of the study, followed by the full dosage from week two onwards. Elderly individuals may be more prone to gastrointestinal sensitivity, and the chosen dosages minimise the risk of side effects, while in keeping with dosages for anticipated brain health effects.

##### 2.7.3.1 Interventional product 1: chicory inulin

The chicory inulin powder is a commercially available powder from Sensus (the Netherlands). The powder is a native inulin, extracted from chicory roots. The agglomerated powder has an excellent dispersibility and wettability. The product has a neutral taste and is readily dissolved in water. Previous studies conducted that had positive effects on cognition include dosages of 5 g (19, 23), 10 g (22) and 16 g (21) per day. Effects on gastrointestinal microbiota were observed at dosages from 2.5 g to 20 g (176–178). A dose of 12 g/day is chosen which is approximately in the middle of the doses that found an effect on cognition. No adverse effects were shown at this dose, but mild flatulence can occur for some individuals (147, 179).

##### 2.7.3.2 Investigational product 2: resistant dextrin

The resistant dextrin powder is commercially available from Roquette (France). The product is a low viscosity soluble fibre, made from wheat through a highly regulated dextrinization process accompanied by chromatographic fractionation (180). The dextrin is poorly digested and absorbed in the ileum, and thus largely available for colonic fermentation (72, 161). The product has a neutral taste and is readily dissolved in water. Gastrointestinal tolerance has been shown at high doses (161). Additionally, the effects of the investigational product in a compote (pea protein, hydrolyzed wheat gluten) was evaluated in elderly (70–90 years), with good digestive comfort and gastrointestinal tolerance (181).

##### 2.7.3.3 Investigational product 3: seaweed *β*-glucan extract

The seaweed investigation product is a non-commercially available product from Oceanium (Scotland). The product consists of a *β*-glucan and fucoidan mixture, isolated from brown seaweed (*Laminaria saccharina,* sugar kelp). Purity assay analyses reported the supplement to contain 66.3% β-glucan, 9.24% fucoidan, 10.27% protein, 2.28% ash and 1.49% alginate on average. Preparation of β-glucan and fucoidan was performed by water-based extraction methods without chemical modification. *Saccharina latissima* (Linnaeus) is one of the dominant kelp-forming species of brown macroalgae in Europe. The seaweed for the product is sustainably farmed in the north Atlantic (182). The seaweed product is fully dissolvable in water. The product has a beige/brown appearance with a toasty/malty odour and taste. The chosen fucoidan dosage has been established as safe (175). Additionally, previous studies of 250 mg/day of *β*-glucan demonstrated effects on mood (24, 25). To ensure volumetric and caloric equality with the placebo arm, 3.5 g twice a day (7 g/day) of placebo (maltodextrin) has been added to the product.

##### 2.7.3.4 Investigational product 4: placebo: maltodextrin

Maltodextrin (Roquette, France) will be used as the placebo in this study. Maltodextrin is frequently used as placebo in human studies, and has been used as placebo in inulin, RD and β-glucan studies before (88, 137, 138). The maltodextrin product is produced by hydrolysis, purification and spray-drying of edible maize starch. The chosen placebo is a short-chain maltodextrin (dextrose equivalent of 18.0–19.9), allowing for readily absorption in the small intestine, and thus virtually eliminates potential prebiotic or fermentative effects in colon. The product is a white powder and has a neutral/slightly sweet taste.

#### 2.7.4 Assignment of interventions

##### 2.7.4.1 Randomisation, stratification, allocation concealment, and blinding

Individuals eligible for participation will be randomly allocated to one of the four study arms by an independent researcher. Participants will be stratified based on: (i) Sex (male vs. female); (ii) LIBRA cognitive risk factor profile (medium risk: 2–3 points vs. high risk: ≥4 points); (iii) Combined age and education level (≥70 years or low education vs. 60–69 years with high education). High education is defined as *HBO associate degree* (Dutch: HBO; Hoger Beroepsonderwijs).

Study arms will be block randomised within each stratum using SAS (183) to ensure a balanced number of participants per study arm. To preserve blinding, each intervention group will be assigned a unique, randomly generated 4-digit code. A designated staff member, not involved in data analysis, will access group allocations to prepare the intervention materials. Supplements and placebo will be packaged in identical, non-transparent sachets to prevent identification. Breaking the randomisation code of an individual participant will be only be permitted in the case of a suspected unexpected serious adverse reaction, serious adverse event (SAE), or other medical indication as determined by a physician.

##### 2.7.4.2 Strategies to enhance adherence and ensure intervention safety

The four investigational products are commercially available, food-grade, prepared in a food-grade environment, and safe for oral consumption. Persons who are allergic to any of the products are excluded from participation. Mild gastrointestinal side effects may occur due to dietary fibre supplementation, which will reduce over time. However, our chosen dosages are in accordance with previous studies indicating gastrointestinal tolerance. To reduce potential side effects and improve compliance, dosage of the fibres are halved and will be tapered-up, as described in the preceding section. Adherence will be monitored by compliance diaries, returned supplement counts at end study visits, and five phone calls throughout the study that will also serve to address any issues that arise.

##### 2.7.4.3 Criteria for discontinuing or modifying allocated interventions

Participants are free to withdraw from the study for any reason and at any time. Investigators may withdraw a subject from the study for urgent medical reasons. Subjects will not be replaced after withdrawal, and a 15% drop-out rate is included in the sample calculation. The study will be prematurely terminated if the medically responsible investigator determines that a SAE might be related to the study, or a reassessment of risk and benefits is deemed necessary. If the study terminates abruptly, data collected up to the date of termination will still be analysed and results published in adherence to the Dutch “Central Committee in Research Involving Human Subjects” (CCMO) guidelines. The Medical Research Ethics Committee (MREC) and participants will be informed as soon as possible about the premature termination. Follow-up of participants will only take place in case of withdrawal for medical reasons.

##### 2.7.4.4 Adverse events

All adverse events reported spontaneously by the subject or observed by the research staff will be recorded. All SAEs will be evaluated by the research physician and reported to the accredited MREC, within 7 days of first knowledge. The study may be prematurely terminated if the participant’s health or safety is under risk.

### 2.8 Outcome measurements

An overview of all questionnaires and outcome assessment markers can be found in Tables 3 and 4.

#### 2.8.1 Brain health markers

##### 2.8.1.1 Functional magnetic resonance imaging

The primary outcome will be evaluated at *T*_0_ and *T*_1_. Working memory function will be assessed using a digit 2-back task during fMRI. Prior studies have demonstrated differential blood BOLD responses in individuals with SCD compared to healthy controls during n-back task performance (117). This validated paradigm consists of three load conditions (0-, 1-, and 2-back), each presented in ten 30-s blocks, with a total task duration of 15 min. A continuous series of single-digit numbers will be shown and participants are instructed to identify whether the current digit matches the one presented (*n*) number of trials earlier. The task includes three working memory load conditions: 0-back (indicated by “–”), in which participants respond when a predefined target digit appears; 1-back (“<−”), in which they respond when the current digit matches the one presented immediately before; and 2-back (“<− −”), requiring detection of matches two positions back in the sequence. Primary outcomes of this study are hence (i) differential BOLD signal responses during the n-back working memory task and (ii) n-back task performance. Analysis of 1-back versus 0-back and 2-back versus 0-back contrasts will identify statistically significant brain activations at low and high cognitive loads, respectively. Main regions of interest include (bilateral) hippocampi, fusiform gyri and dorsolateral prefrontal cortices, based on their previous association with working memory (184, 185). Anatomical MRI scans (*T*_1_-weighted and FLAIR) will be used to assess total/regional grey matter and white matter integrity (hyperintensities via Fazekas scale or automated volumetry), focusing on the hippocampi and (pre)frontal- and temporal cortices as regions of interest.

##### 2.8.1.2 Cognitive and mood assessments

Cognitive and mood outcomes will be assessed *T*_0_, *T*_mid_ and *T*_1_ visits (Table 3). The neuropsychological test battery (NTB) consists of the Cognitive-Functional Composite (CFC) and the Montreal Cognitive Assessment scale (MoCA) - the latter assessed at *T*_0_ only. The CFC battery provides a short and clinically relevant measurement of the cognitive domains that are sensitive to decline in early AD (186). The brief CFC NTB consists of six cognitive tests focusing on episodic memory, working memory and executive functions (186). Components included within the CFC are three components of the Alzheimer’s Disease Assessment Scale-Cognitive subscale (ADAS-Cog) (word recall, orientation, and word recognition), as well as a digit span test, verbal fluency test, and digit symbol substitution test. These tests can detect subtle worse performance in SCD subjects (112). The MoCA scale is a test with high sensitivity and specificity for minor cognitive defects (if scored ≤26/30) (187).

The Geriatric Depression Scale-15-item version (GDS-15) is a concise tool specifically developed to screen for depression in older populations, with established cut-offs and has demonstrated high sensitivity and specificity in older adults (188). The Generalized Anxiety Disorder-7 (GAD-7), a seven-item questionnaire, validated for use in older adults, will be employed to evaluate the presence of anxiety symptoms in daily life (189, 190). Subjective cognitive decline, will be measured via the Cognitive Failure Questionnaire (CFQ), a 25-item scale quantifying the frequency of everyday cognitive lapses (191, 192).

##### 2.8.1.3 Brain health related blood markers

The *apolipoprotein E (ApoE) genotype* will be measured at *T*_0_ following DNA extraction from whole blood. ApoE genotyping is a well-established genetic risk factor for AD. Carriage of the ε4 allele is an AD risk factor, while the ε2 allele has a protective effect (193). Within the SCD + criteria framework, the presence of the ApoE ε4 allele increases risk for SCD progression to MCI or AD (113). ApoE genotype modulates lipid metabolism, with potential consequences for cognitive function, and will be evaluated alongside lipid profiles (194, 195). Plasma *Aβ1–42/Aβ1–40* ratio, *Neurofilament light chain* (NfL) and *glial fibrillary acidic protein* (GFAP), are emerging AD biomarkers in blood, and will be measured at *T*_0_ and *T*_1_ with single molecule array (SIMOA) analyses (196). The Aβ1–42/Aβ1–40 ratio as a biomarker is strongly correlated with Aβ deposition in the brain, and is predictive of cognitive decline along the AD continuum (197, 198). In SCD, lower plasma ratios were associated with steeper rate of decline on tests for attention, memory, and executive functioning (199). NfL is released into the blood and cerebrospinal fluid (CSF) after axonal injury and is indicative of neuroaxonal degeneration (200). Increased serum levels are associated with cognitive impairment as seen in MCI, and may serve as a prognostic biomarker of AD (200, 201). Similarly, GFAP, a marker of reactive astrogliosis, is increased in preclinical AD and may serve as sensitive biomarker for indicating CSF Aβ pathology (202).

*Serum cortisol* and *brain derived neurotrophic factor (BDNF)* levels will be determined by ELISA at *T*_0_ and *T*_1_. Reduced cortisol levels have been associated with worsened overall cognitive functioning, and with MCI, potentially due to HPA axis alterations and glucocorticoid-induced neurotoxicity (157). *BDNF* is a neurotrophin that regulates neuronal development and function (203). BDNF has neuroprotective properties, modulates synaptic efficacy at both glutamatergic and GABAergic synapses and plays a crucial role in synaptic plasticity, particularly in late-phase long-term potentiation (204, 205). Abnormal BDNF levels have been seen in AD (206).

Neurotransmitters and tryptophan-related metabolites will be measured in serum using ultra-high-performance liquid chromatography with electrospray ionization tandem mass spectrometry (UHPLC-ESI-MS/MS) at *T*_0_ and *T*_1_ (207). Tryptophan metabolites play a central role in MGBA modulation, with metabolites that are neurotoxic (quinolinic acid) and neuroprotective (kynurenic acid), while the kynurenine/tryptophan (KYN/TRP) ratio may serve as a peripheral biomarker of neuroinflammation (159, 160, 208, 209). Several of these metabolites can cross the BBB and modulate cognitive and behavioural processes (210) Furthermore, microbiota derived tryptophan indoles have been associated with improved cognitive outcomes (82, 211). Metabolites from the following pathways will be quantified: Kynurenine pathway; Tryptophan hydroxylation pathway; Microbiota-associated tryptophan metabolites, Tyrosine pathway; Phenylalanine metabolism; Branched chain and other amino acids; Histaminergic system; Microbial-derived methylamines.

#### 2.8.2 Gut health markers

##### 2.8.2.1 Faecal sample collection

Participants will collect faeces at home using a faecal collection paper (Faecesvanger, Tag Hemi VOF, The Netherlands) and a collection container with a collection scoop incorporated in the lid (Sarstedt, Nümbrecht, Germany). Participants will be instructed to collect from three different topographical locations of the faeces, and fill the container until two-thirds. Directly after collection, samples will be stored at −20 °C at home. Samples will be transported under frozen conditions in cooler bags with ice packs to the research facility and immediately stored at −80 °C upon arrival.

##### 2.8.2.2 Gastrointestinal questionnaires

Gastrointestinal symptoms will be assessed using the Gastrointestinal Symptom Rating Scale (GSRS) and the Bristol Stool Scale (BSS). The GSRS includes 15 items across five symptom clusters: abdominal pain, reflux, indigestion, constipation, and diarrhoea, scored from 1 (no discomfort) to 7 (very severe discomfort) (212). It is validated for use in healthy adults and has been applied to assess changes in gastrointestinal symptoms with increased fibre consumption (213). The BSS is a seven-point scale that categorises stool form, with Types 1–2 indicating constipation, Types 6–7 diarrhoea, and Types 3–5 considered normal. It includes pictorial representations and is widely used in clinical and research contexts (214). The GSRS will be completed at all study visits and weekly during the first month. The BSS will be completed with each faecal sample collection.

##### 2.8.2.3 Intestinal transit time

ITT is in an indicator of gut motility and function (215). In addition, ITT is significantly associated with gut microbiota richness and composition (216). Participants will be asked to consume two muffins, containing in total 1.5 gram blue colouring agent (EFSA approved for use in food: E133, E122). Time and date of consumption will be recorded, followed by time of first visualisation of blue/green colour in stool passage on the BSS questionnaire form. ITT can then be calculated (215).

##### 2.8.2.4 Microbiota analysis

Quantification of the total number of bacteria via the total 16S rRNA copy number and quantifications of selected bacteria, such as the main phyla *Bacteroidetes* and *Firmicutes*, and *Bifidobacterium* and *Lactobacillus* species that are expected to change upon intervention, will be determined by droplet-digital PCR (ddPCR). Qualitative microbiota composition will be determined by 16S rRNA gene-based sequencing. Bioinformatic analysis will focus on changes in both richness, diversity and compositional changes. Analysis of the microbiota functional pathways will be carried out in R by Picrust, Tax4fun and KEGG pathway analysis to search for alterations in microbiota activity caused by the interventions (217, 218).

##### 2.8.2.5 Microbiota derived metabolites

Short chain fatty acids (acetate, propionate, butyrate, valerate) as well as branched chain fatty acids (iso-valeric acid, iso-propionic acid) will be measured in plasma (LC–MS/MS; liquid chromatography tandem mass spectrometry) and faecal samples (GC-FID; gas chromatography-flame ionization detection) (219).

##### 2.8.2.6 Intestinal function

Ageing impairs gut barrier function leading to increased permeability and systemic inflammation (38, 220). Biomarkers such as intestinal fatty-acid binding protein (iFABP) indicate epithelial damage, while LPS-binding protein (LBP) and soluble cluster of differentiations 14 (sCD14) reflect immune activation following LPS translocation (221). Zonulin regulates tight junctions (222), while elevated calprotectin and lipocalin indicate intestinal inflammation (223–226). Ageing is also associated with reduced mucosal thickness and soluble immunoglobulin A (sIgA) expression, compromising mucosal defenses (33, 35). Water content is a more reliable marker of stool consistency than questionnaire forms, while faecal pH indicates faecal SCFA levels (219). Faecal water content and pH (219), faecal intestinal inflammatory markers (Calprotectin, Lipocalin-2, *β*-defensin, sIgA), plasma/serum intestinal integrity markers (iFABP, LBP, sCD14) and tight junctional integrity (Zonulin) will be analysed by commercially available ELISAs.

#### 2.8.3 Metabolic and inflammatory markers

Increased low-grade, systemic inflammation is seen with ageing (227) and may lead to neuroinflammation (228) and neurodegenerative processes (229). *Systemic inflammation* will be measured using an O-link cytokine panel (96 inflammatory markers) (230). Vitamin D insufficiency is common amongst older adults in Northern Europe and has been linked to inflammation, intestinal dysfunction, and cognitive decline (231, 232). *Serum vitamin D* will be assessed by the KCHL laboratory, ZGV. Insulin resistance is a known predictor of cognitive decline (158), while abnormal total cholesterol and HDL levels have been associated with increased risk of cognitive impairment (233, 234). *Metabolic markers* (fasting glucose, insulin, and HbA1c) as well as a *lipogram* (HDL, LDL, total cholesterol, triglycerides) will also be analysed at KCHL laboratory.

#### 2.8.4 Dietary assessments

Dietary intake will be assessed by means of a food-frequency questionnaire (FFQ), which has been adapted for ease of calculation of a Mediterranean-DASH diet Intervention for Neurodegenerative Delay (MIND)-diet score (235). The MIND-NL score has been tailored to the Dutch context by incorporating commonly consumed Dutch food products, converting serving sizes to weights or volume amounts. The MIND diet dietary pattern has been designed as a preventative dietary pattern for cognitive decline, and higher adherence has been shown to be associated with protective effects on global cognition and lowered dementia risk (236). The FFQ will additionally be used to determine habitual dietary patterns and fibre intake.

#### 2.8.5 Urine analysis

Urine will be collected at *T*_0_ and *T*_1_ for metabolomic analysis related to microbiota metabolites and neurotransmission.

#### 2.8.6 E-health measures

Samsung Active 2.0 smartwatches will be used to measure heart rate, physical activity and mood. Physical activity is measured using both the pedometer (step count) and accelerometer data. The software has been modified in accordance with the protocol used in the DiaGame Study (Maxima Medical Center, Eindhoven and the Eindhoven University of Technology) (237). This software modification incorporates a push notification asking the participant to record their mood from five provided options (sad, stressed, neutral, happy, and angry) using the Experiencer application (238). Push notifications will be sent at random throughout the day, with a cooldown period set to every 3 h to ensure participant engagement. A previous study indicated that there is a meaningful congruency with longer depression and anxiety questionnaires (239). Step count is recorded continuously in an accumulative manner, with data points logged whenever new pedometer data becomes available. Accelerometer and heart rate data are captured when the user engages with the watch during self-reports. Data from the heart rate monitor will be used to determine the resting heart rate, as well as being used to infer the frequency, duration, and intensity of periods of increased physical activity/exercise. For each feature, the mean and variance across the 7 days will be calculated. To validate the mood data gathered by the smartwatches, data will be compared to validated depression and anxiety questionnaires. Once the device has been returned to WUR, the data will be extracted and all collected data will be securely uploaded through the Application Programming Interface (API) of GameBus platform which controls and processes them according to the General Data Protection Regulation (GDPR) and the Dutch Act on Implementation of the GDPR. The data will be stored on secure GameBus servers during the study and research analysis and will be removed after the analysis and termination of the study (240).

### 2.9 Statistical methods, data collection and management

#### 2.9.1 Statistical methods for primary and secondary outcomes

Data analyses will be performed using R-studio, GraphPad and SPSS. Numerical values will be reported as mean ± SD or confidence intervals. The level of significance is set at 0.05 (two-sided). The Benjamini-Hochberg correction will be used to correct and adjust all *p*-values for multiple comparisons, with the inclusion of a false discovery rate.

The *primary objective* of this study is to investigate the effect of 26 weeks of supplementation with three different dietary fibres (chicory inulin, resistant dextrin, and seaweed polysaccharide) compared to a placebo on working memory (fMRI). Analyses for the primary outcome will compare each fibre group individually to placebo using ANCOVA to adjust for baseline differences, and evaluate the effect of the intervention group at 26 weeks. Specifically, averaged beta weights will be extracted from the regions of interest to determine significant activation at low cognitive load (1-back versus 0-back) and high cognitive load (2-back versus 0-back). *d*-prime values will be calculated to assess working memory task performance. Linear mixed models will be used to evaluate effects on the *secondary and tertiary outcomes* of each dietary fibre individually compared to the placebo at the two different timepoints (baseline and week 26) to measure the interaction group x time. Participants will be set as the random factor, with group and time set as the non-random (fixed) factors. Post-hoc analyses will be performed in case of a significant group x time interaction to understand which fibre(s) specifically differ from the placebo group.

Additionally, *exploratory analyses* will evaluate whether gut- or brain-related biomarkers (e.g., kynurenic acid, serotonin, tryptophan indoles) are associated with cognitive (NTB, MoCA), mood (GDS-15, GAD-7), or physiological outcomes. To elucidate potential mechanisms of the dietary fibre intervention along the MGBA, mediating effects of SCFA levels, TRP metabolite levels and intestinal barrier markers (e.g., LBP, zonulin) between fibre intervention group and cognitive outcomes will be evaluated in case of significant main effects.

In statistical analyses, sex, age, and ApoE genotype will be taken into account as potential modifyers. Furthermore, baseline characteristics such as microbiota composition, habitual fibre intake, risk factor profile and baseline cognitive level may be considered as relevant modifyers determining responsiveness to the dietary fibre intervention.

#### 2.9.2 Data management and confidentiality

Data from study visits, measurements and questionnaires will be captured online in CASTOR EDC (127) and verified by two researchers where possible. Participants will receive a unique identification number, used to label all collected data and biological samples. Published data will not be traceable to the identification number. Personal details will be stored separately in secure digital file. Signed informed consent forms and printed questionnaires will be stored in a secure locker. Personal data and biological material, will be retained for 15 years following study completion, after which it will be destroyed. The handling of personal data will comply with the EU GDPR and the Dutch Act on Implementation of the GDPR.

#### 2.9.3 Dissemination

Finding from this trial will be published in accordance with the CCMO’s publication statement. All parties within the study consortium have agreed upon the CCMO directive. Findings from this trial will be disseminated through peer-reviewed, open access publications, conference presentations, and submission to relevant trial registries and databases. Results will be shared with participating institutions and funding partners. Neither the subsidising parties, nor the principal investigator has a right of veto regarding the way of publishing the results. Partners have the right to take cognizance of the results in order to patent some of the outcomes if applicable

## 3 Initial results

By July 2025, baseline measurements were successfully completed. In total, 151 volunteers were randomized into one of four arms and started the intervention. The mean age of our population was 69.4 years (SD = 4.8) and the population consisted of 47.7% females (*n* = 72). The average number of modifiable risk factors was 3.05 (SD = 0.93) and the mean BMI in the population was 27.91 kg/m^2^ (SD = 3.87). Most of the participants (55.6%, *n* = 84) were highly educated, as defined by having at least a 2-year associate degree at a University of Applied Sciences [in Dutch: Hoger Beroeps Onderwijs (HBO)]. Of all subjects, 37.7% carried one or more APOE ε4 allele. The mean MoCA score was 26.09 out of 30 (SD = 2.30) and the average habitual dietary fibre intake was estimated to be 23.68 grams per day (SD = 8.22). Baseline characteristics per intervention arm are presented in Table 6.

**Table 6:** Baseline characteristics.

		Total	Intervention group			
			A	B	C	D
		*n* = 151	*n* = 37	*n* = 38	*n* = 38	*n* = 38
Sex	% female	47.7	45.9	47.4	50.0	47.4
Education level	% 2-year HBO associate degree or higher	55.6	59.5	44.7	47.4	71.1
APOE genotype	% ε4 carrier	37.7	45.9	39.5	39.5	26.3
Age	mean (sd)	69.4 (4.8)	69.9 (4.7)	69.1 (4.6)	68.5 (4.4)	69.9 (5.6)
Number of risk factors	mean (sd)	3.05 (0.93)	3.03 (0.83)	3.03 (0.85)	3.11 (0.98)	3.03 (1.08)
BMI (kg/m^2^)	mean (sd)	27.91 (3.87)	28.0 (3.88)	28.0 (4.78)	28.2 (3.47)	27.5 (3.30)
Habitual fibre intake (g/day)	mean (sd)	23.68 (8.22)	22.76 (9.29)	23.08 (7.92)	23.91 (6.61)	24.99 (8.94)
MoCA score (max 30)	mean (sd)	26.09 (2.30)	26.16 (2.36)	25.55 (2.81)	26.45 (1.93)	26.18 (2.00)

APOE, Apolipoprotein E; SD, standard deviation; BMI, body mass index; MoCA, Montreal Cognitive Assessment.

## 4 Discussion

The PRECODE study will provide an in-depth evaluation of the microbiota-gut-brain axis in older adults, at risk of cognitive decline. Through targeting modulation of the gut microbiota and metabolites by dietary fibres, this study aims to evaluate potential protective effects in SCD+. Repeated failures of disease-modifying treatments in age-related neurodegenerative conditions have highlighted the urgent need to prioritise prevention over late-stage intervention. The study hereby addresses knowledge gaps needed for advancement of preventative strategies.

The study population of individuals with SCD + and additional life-style (LIBRA-score) risk factors, is ideally suited as a target cohort to evaluate potential protective roles on cognition, as individuals are by definition clinically unimpaired. SCD is considered a compensatory phase in preclinical AD, where neural compensation mechanisms may mask functional deficits, and subtly worse performance in neuropsychological tests are seen compared to healthy controls (112–114, 117). The study design allows for the assessment of these subtle cognitive deficits, as both the primary outcome measure, 2-back task during fMRI scanning, and secondary outcome NTB subscales, are sensitive to detect these early cognitive changes (112, 117). Additionally, the 26-week duration of the intervention aligns with previous studies demonstrating significant effects on memory in SCD (241).

The investigation of this cohort is further strengthened by the measurement of AD-related markers, such as ApoE4 ε4 genotype and plasma Aß 41/42 ratios, which indicate a further increased risk of progression to AD (113, 114). Additionally, GFAP and NfL measurements, indicative of neuroglial reactivity and neuraxial injury, respectively (200, 202), will offer insights into potential neuroprotective mechanisms of fibre supplementation, and may provide novel understandings of the role of these emerging biomarkers along the MGBA. Baseline characterisation will allow for subgroup analyses in ApoE ε4 -carriers vs. non-carriers. Recent cross-sectional studies suggest differing cognitive protective effects of dietary fibre based on ApoE-genotype (242). Our study may further elucidate genotype-specific dietary fibre effects along the MGBA, that may pave the way for personalised nutrition approaches in high-risk individuals.

A further major strength of the PRECODE study design lies in the multidomain, mechanistic nature. In-depth exploration of multiple outcomes integrating neuroimaging, microbial, metabolic, and cognitive endpoints, allows for a more complete characterisation of MGBA pathways. Notably, this provides a unique opportunity to investigate associations between gut-derived changes and both functional and structural brain effects, as neuroimaging-omics approaches are lacking in human preclinical AD research (243, 244). PRECODE enables correlation analyses between gut microbial composition, microbial metabolites (SCFAs, indoles), and brain outcomes such as functional connectivity, vascular response, and regional brain volume, within the context of working memory performance. Biomarkers such as BDNF, cortisol, and inflammatory cytokines will support investigation into systemic mechanisms linking the gut and brain. Furthermore, measurements of glycaemic control and insulin resistance, which predict cognitive decline, will allow examination of metabolic pathways contributing to neuroprotection (158, 245).

Targeted metabolite analysis of SCFAs and tryptophan-derived indoles, further strengthens mechanistic insights into how microbiota-derived compounds may modulate brain health. The use of multiple biomatrices, such as SCFA measurement in both blood and faeces, will allow for quantification of systemic absorption and may provide more precise associations with cognitive outcomes. Favourable indoles, such as IPA and indole lactic acid, display potent antioxidant and anti-inflammatory properties and may inhibit Aβ aggregation (82, 211, 246). These effects are partly mediated through activation of the immunomodulatory aryl hydrocarbon receptor (AhR) receptor, a regulator of neuroinflammation (247–249). Preclinical studies suggest that AhR activation by indoles promotes youthful gene expression profiles, and extends healthy lifespan (250). PRECODE may thus provide novel insights into the potential protective roles of these metabolites in early cognitive ageing and healthspan promotion, supporting their investigation as candidate biomarkers. In addition, distinct microbial and metabolite signatures may be identified, that can inform on mechanistic pathways as well as cohort classification in preclinical AD.

The inclusion of a mid-study visit after 13 weeks, allows for determining the duration of intervention needed for microbiota and metabolite shifts. Furthermore, mid-study effects on cognition by NTB will allow for insights into timing of onsets of potential protective cognitive effects. Moreover, sex, ApoE genotype and age, influence AD disease stage progression (251). Here, further valuable information on whether these factors may influence the trajectory of potential interventions may be highlighted. Moreover, should there be any significant association evidenced between microbial compositional change and cognitive performance, it might be worthwhile to conduct a follow-up investigation, in which we research if and how long potential microbiota changes last after the trial has been finished, and to compare E4 vs. non-E4 carriers and relatedness of the number of LIBRA risk factors.

Despite its strengths, the study has potential limitations. The self-reported and progressive nature of SCD + may introduce heterogeneity, such as the potential inclusion of individuals with undiagnosed depression and anxiety that may mimic cognitive decline (114, 252). Clinical depression and anxiety are excluded at screening, and will be further assessed by GDS-15 and GAD-7 scores at baseline. Furthermore, some individuals may potentially convert to amnestic MCI over the course of the intervention, which will be evaluated by end-NTB results. Inclusion criteria, such as requirements for general and technological literacy, may additionally introduce bias towards individuals with higher education or better cognitive function. In addition, the inclusion criterion of at least 2 LIBRA risk factors next to presence of SCD may overall limit the generalizability of our findings to SCD individuals with fewer or different combinations of lifestyle risk factors, and should not be generalized to the entire preclinical AD spectrum. Next, long-term fibre supplementation in older adults with increased gastrointestinal sensitivity, may lead to study withdrawal, for which a 15% drop-out rate has been included. Strategies such as dose tapering, morning and evening dosing, and telephone check-ins are used to promote adherence. Furthermore, due to the scope of inclusion and exclusion criteria, including MRI compatibility, extensive recruitment efforts might be needed.

To our knowledge, PRECODE is the first human study to investigate the combined cognitive and microbial effects of three distinct dietary fibres- chicory inulin, resistant dextrin and seaweed polysaccharide, in SCD+. The extensive mechanistic and functional outcomes will provide valuable insights into the role of the MGBA in preclinical cognitive decline, advancing the field of nutrition-based prevention in cognitive ageing and contributing toward the development of personalised strategies in preclinical AD.

## Glossary

5-HT: 5-Hydroxytryptamine
Aβ: Amyloid beta
AD: Alzheimer’s Disease
ADAS: Alzheimer’s Disease Assessment Scale
AhR: Aryl Hydrocarbon Receptor
API: Application Programming Interface
ApoE: Apolipoprotein E
BBB: Blood–Brain Barrier
BCFA: Branched Chain Fatty Acid
BDNF: Brain Derived Neurotropic Factor
BMI: Body Mass Index
BOLD: Blood Oxygenation Level Dependant
BSS: Bristol Stool Scale
CCMO: Central Committee on Research Involving Human Subjects; *in Dutch: Centrale Commissie Mensgebonden Onderzoek*
CFC: Cognitive-Functional Composite
CFQ: Cognitive Failure Questionnaire
CSF: Cerebrospinal Fluid
(dd)PCR: (Droplet Digital) Polymerase Chain Reaction
EDC: Electronic Data Capturing
EFSA: European Food Safety Authority
ELISA: Enzyme-Linked Immunoassay
FFQ: Food Frequency Questionnaire
FLAIR: Fluid-Attenuated Inversion Recovery
(f)MRI: (Functional) Magnetic Resonance Imaging
GABA: Gamma-Aminobutyric Acid
GAD-7: Generalised Anxiety Disorder-7
GC-FID: Gas Chromatography-Flame Ionization Detection
GDPR: General Data Protection Regulation
GDS-15: Geriatric Depression Scale-15
GFAP: Glial Fibrillary Acidic Protein
GPR: G-Protein Coupled Receptor
GSRS: Gastrointestinal Symptom Rating Scale
HbA1c: Glycated haemoglobin
HBO: Dutch: Hoger Beroepsonderwijs
HDAC: Histone Deacetylase
HDL: High Density Lipoprotein
HPA: Hypothalamic Pituitary Adrenal
iFABP: Intestinal Fatty Acid Binding Protein
IL: Interleukin
IPA: Indole Propionic Acid
ILA: Indole Lactic Acid
ISAPP: International Scientific Association for Probiotics and Prebiotics
ITF: Inulin-Type Fructan
ITT: Intestinal Transit Time
KYN: Kynurenine
LBP: LPS-binding protein
LC–MS: Liquid Chromatography–Mass Spectrometry
LDL: Low Density Lipoprotein
LIBRA: LIfestyle for BRAin health criteria
LPS: Lipopolysaccharide
MCI: Mild Cognitive Impairment
MREC: Medical research ethics committee (MREC)
MGBA: Microbiota-Gut-Brain-Axis
MIND: Mediterranean-DASH Diet Intervention for Neurodegenerative Delay
MoCA: Montreal Cognitive Assessment scale
NfL: Neurofilament light chain
NTB: Neuropsychological Test Battery
PTSD: Post traumatic stress disorder
RD: Resistant Dextrin
ROI: Region of Interest
ROS: Reactive Oxygen Species
rRNA: Ribosomal Ribonucleic Acid
SAS: Statistical Analysis System
SCD (+): Subjective Cognitive Decline (Plus)
sCD14: Soluble Cluster of Differentiation 14 Subtype
SCFA: Short Chain Fatty Acid
SAE: Serious Adverse Event
sIgA: Secretory Immunoglobulin A
SIMOA: Single Molecule Array
TICS-m: modified Telephonic Interview for Cognitive Screening
TNF-a: Tumour Necrosis Factor Alpha
TRP: Tryptophan
UHPLC-ESI-MS/MS: Ultra-High Performance Liquid Chromatography-Electrospray Ionization Tandem-Mass Spectrometry
WMO: Medical Research Involving Human Subjects Act; *in Dutch: Wet Medisch-wetenschappelijk Onderzoek met Mensen*
WUR: Wageningen University and Research
ZGV: Ziekenhuis Gelderse Vallei (*Gelderse Vallei Hospital*)
